# The effect of social participation on income-related inequality in health outcome among Chinese older adults

**DOI:** 10.1093/inthealth/ihaa023

**Published:** 2020-06-02

**Authors:** Jian Sun, Xiaoyin Lyu, Shoujun Lyu, Rui Zhao

**Affiliations:** School of International and Public Affairs, Shanghai Jiao Tong University, Shanghai, China; High School Affiliated to Shanghai Jiao Tong University, Shanghai, China; School of International and Public Affairs, Shanghai Jiao Tong University, Shanghai, China; China Institute of Urban Governance, Shanghai Jiao Tong University, Shanghai, China; Affiliated Hospital of Hebei University, Baoding, Hebei, China

**Keywords:** social participation, income-related inequality, health outcome, older adults, China

## Abstract

**Background:**

This study aimed to investigate the effect of social participation on income-related inequality in health outcome among older adults in China.

**Methods:**

The panel data used in this study were sourced from the 2011 and 2014 waves of the Chinese Longitudinal Healthy Longevity Survey (CLHLS). Furthermore, this study employed a concentration index to assess the income-related inequality in health outcome. Moreover, this study used the decomposition method of concentration index to analyse the effect of social participation on income-related inequality in health outcome.

**Results:**

The total concentration index of Instrumental Activity of Daily Living (IADL) status decreased from 0.0257 in 2011 to 0.0172 in 2014. Furthermore, the total concentration index of psychological health decreased from 0.0309 in 2011 to 0.0269 in 2014. The decomposition analysis indicates that social participation made a major contribution to the pro-rich inequality in IADL status. Moreover, the results also indicate that social participation made a minor contribution to the pro-rich inequality in psychological health.

**Conclusions:**

This study demonstrated that overall there were pro-rich inequalities in IADL status and psychological health among older adults in China. Moreover, social participation made a major contribution to the pro-rich inequality in IADL status, while it made a minor contribution to the pro-rich inequality in psychological health.

## Introduction

The Chinese population has been aging rapidly over the past several decades. The number of older adults ≥65 y of age increased from 88.11 million in 2000 to 166.58 million in 2018 and its proportion in the total population rose from 6.96% to 11.90%.^1,2^ It is estimated by the World Health Organization (WHO) that the number of Chinese adults ≥65 y of age will increase to >300 million and account for approximately 30% of the total population by 2050.^3^ One study indicated that China will become the fastest aging large nation in the course of the first half of the 21st century.^4^ Previous studies indicate that an increase in the proportion of the elderly population can increase healthcare expenditures^5,6^ and population aging is still a crucial challenge.^7^ Furthermore, there is a higher prevalence of chronic diseases and functional disability among older adults. In 2013, >100 million older adults had chronic non-communicable diseases in China.^8^ At the same time, >37 million older people had disabilities in physical function.^8^ Consequently, population aging is challenging the systems of social security and healthcare.^9^ In 2016, the Healthy China 2030 Strategy Planning Outline was issued by the Chinese government, which pointed out that the government intends to prioritize population health.^10^

Social capital can be divided into structural social capital and cognitive social capital. Structural social capital refers to the actual participation in various social networks, while cognitive social capital refers to perceptions about social network involvement.^11^ Social participation is a core aspect of structural social capital.^12^ Previous studies indicate that social participation is determined by age, gender, education status, marital status, household income and residency location, among others.^13,14^ Furthermore, social participation profoundly affects people's lives and plays an important role in integrating people into society. Older adults’ social participation has been regarded as an important part of active aging or successful aging.^15^ In 2002, the United Nations (UN) put forward the policy framework of active aging to deal with population aging in the 21st century. The Chinese government also adopted an active aging policy to achieve successful aging. In November 2019, the Chinese government issued National Medium and Long Term Planning on Active Responding to Population Aging, which stated that active responding to population aging is a major national strategy and it is necessary to perfect health services system to promote the physical and mental health of the elderly.

Previous studies have explored the impact of social participation on health outcomes among older adults. Glass et al.^16^ found that social participation plays a vital role in reducing mortality among the elderly. Some studies have indicated that social participation can significantly promote self-rated health.^17–21^ A longitudinal study in Japan suggested that social participation can slow functional decline among the elderly.^22^ Furthermore, some studies indicated that social participation can enhance cognitive health and protect against cognitive decline among older adults.^23,24^ Croezen et al.^25^ used data from the Survey of Health, Ageing and Retirement in Europe (SHARE) and found that social participation can reduce depression in the elderly. A study in China also discovered that participation in social activities decreased the risk of suffering from depressive symptoms.^26^

Overall, these studies have examined the health effect of social participation, but few studies have focused on the relationship between social participation and income-related inequality in health outcomes. To fill this gap, this study aimed to investigate the effect of social participation on income-related inequality in health outcomes among older adults in China. The results of this study could provide a deep understanding of the effects of social participation on income-related inequality in health outcomes among Chinese older adults.

## Methods

### Data source

The panel data used in this study were sourced from the 2011 and 2014 waves of the Chinese Longitudinal Healthy Longevity Survey (CLHLS). The CLHLS is the largest national longitudinal survey of adults ≥65 y of age in mainland China. Furthermore, it was conducted by the Center for Healthy Aging and developed at Peking University and provides information on health status and quality of life of the elderly in 22 provinces of China. The aim of CLHLS is to shed light on the determinants of healthy human longevity and oldest-old mortality. The CLHLS was performed in 1998, 2000, 2002, 2005, 2008–2009 (late 2008 and early 2009), 2011–2012 (late 2011 and early 2012) and 2014. The CLHLS randomly selected half of the counties in 22 of 31 Chinese provinces, covering about 85% of the total population in China.^27^ The CLHLS data are high quality and many scholars use the survey for academic research.^27–31^ Given the fact that the CLHLS provides rich information concerning health outcomes, social participation, health-related behaviour, cognitive ability, demographic characteristics and socio-economic status of older adults, this study used it to examine the effect of social participation on income-related inequality in health outcomes. We deleted the observations with abnormal and missing values. After data cleaning, a total of 2366 participants ≥65 y of age from the 2011 and 2014 waves of the survey, including 4732 observations, were included in this study.

### Variables

#### Dependent variables

Health is a multidimensional concept, including physical and psychological health. In this study we used physical and psychological health to measure health outcomes. Physical health was measured by the Instrumental Activity of Daily Living (IADL) scale, which was developed by Lawton in 1973.^32^ The IADL scale measures the ability to carry out outside activities and use instruments in daily lives for the elderly. In this study, IADL status was assessed by whether a respondent had difficulty in performing eight activities that are quite important for independent living: visiting neighbours, shopping, cooking, washing clothes, walking 1 km, carrying 5 kg, crouching and standing three times and taking public transportation. The response for each question was ‘yes’, ‘a little difficult’ or ‘unable to do so’. We categorized respondents as IADL disabled (coded as 0) if they had any difficulty in performing any of the eight items (otherwise coded as 1).

In addition, psychological health was measured by presenting respondents with five statements. Among the five statements, two were positively oriented: ‘I look on the bright side of things’ and ‘I am as happy as younger people’. Three of the statements were negatively oriented: ‘I feel fearful or anxious’, ‘I feel lonely and isolated’ and ‘I feel useless with age’. All of the questions had five response levels: ‘always’ (coded as 1), ‘often’ (coded as 2), ‘sometimes’ (coded as 3), ‘seldom’ (coded as 4) and ‘never’ (coded as 5). We reverse-coded the positively oriented questions and the older adults’ psychological health was assessed based on a 5-point Likert-type scale (‘never’ coded as 0, ‘seldom’ coded as 1, ‘sometimes’ coded as 2, ‘often’ coded as 3 and ‘always’ coded as 4). The scores from the five statements were summed to get a score for each older adult, ranging from 0 to 20, with higher scores indicating better psychological health.

#### Independent variable

In this study, social participation is the independent variable. In the CLHLS, respondents were asked to answer whether they engaged in social activities and their answers were dichotomized into ‘yes’ (1) and ‘no’ (0).

### Control variables

Previous studies revealed that demographic characteristics, socio-economic status and health-related behaviour affected income-related inequality in health outcomes, thus we selected them as control variables.^26,33^ In this study, the first type of control variable described the demographic characteristics, including age, gender (male coded as 1), marital status (married coded as 1) and residency location (living in urban area coded as 1). The second type of control variable described the socio-economic status, including years of schooling (continuous variable), household income (continuous variable) and health insurance (having health insurance coded as 1). In addition, in order to reduce heteroscedasticity, we used the log-transformed household income in regression models. The third type of control variable described the health-related behaviour, including physical exercise (taking part in physical exercise coded as 1).

In addition, this study performed a Variance Inflation Factor (VIF) test. The test shows that the mean VIF is 1.29 and the VIF values of independent variables and control variables are far lower than the critical value of 10, which indicates that there was no serious multicollinearity across the regression models.

### Methodology

The concentration index is defined as twice the area between the concentration curve and the line of equality (the diagonal).^34^ Income-related inequality in health outcome can reflect good health outcome is concentrated among higher-income people or lower-income people. Given that the concentration index is considered to be a useful tool to estimate the degree of inequality in health outcomes caused by socio-economic factors,^35,36^ this study employed it to assess income-related inequality in health outcomes among the elderly. Furthermore, the concentration index ranges between −1 and +1, where a positive value indicates that the health outcome variable is more concentrated among the higher-income people, and vice versa.^37–40^ If there is no income-related inequality in health outcome, the concentration index will equal zero.^41^ Moreover, the absolute value of the concentration index is larger, indicating a greater degree of income-related inequality in health outcome.^42^ In addition, the formula used for calculating the concentration index can be written as:

(1) \begin{equation*}C = \frac{2}{\mu }{\mathop{\rm cov}} ({y_i},{r_i}i),\end{equation*}

where *C* is the concentration index, *μ* is the mean of health outcome indicator, *y_i_* is the health outcome indicator and *r_i_* is the fractional rank of household income.

The panel data model can address the endogeneity caused by unobservable individual heterogeneity, allowing us to correctly understand the relationship between variables. We used it to investigate the effect of social participation on health outcomes among older adults. Furthermore, panel data models include random effects models and fixed effects models, and we used the Hausman test to determine which model to use. Moreover, considering the fact that IADL status is a dichotomous variable, a panel Logit regression model was employed to explore the impact of social participation on IADL status among Chinese older adults. The panel Logit regression model is as follows:

(2) \begin{equation*}Ln\left( {\frac{{{P_{it}}}}{{1 - {P_{it}}}}} \right)\ = {\alpha _0} + {\alpha _1}*S{P_{it}} + {\alpha _2}*C{V_{it}} + {\varepsilon _{it}}\end{equation*}

where *i* is the individual, *P_it_* is the possibility of having no IADL problem for individual *i* in period *t, α*_0_ is the intercept term and *α*_1_ and *α*_2_ are the regression coefficients for social participation and control variables, respectively. In addition, *SP_i__t_* is the social participation, *CV_i__t_* is the control variables and *ε_i__t_* is the error term.

In addition, since psychological health was a continuous variable, the panel linear regression model was adopted to investigate the effect of social participation on psychological health. The panel linear regression model is as follows:

(3) \begin{equation*}P{H_{it}} = {\beta _0} + {\rm{ }}{\beta _1}*S{P_{it}} + {\rm{ }}{\beta _2}*C{V_{it}} + {\varepsilon _{it}},\end{equation*}

where *PH_it_* is the psychological health for older adult *i* in period *t, β*_0_ is the intercept term and *β*_1_ and *β*_2_ are the regression coefficients for social participation and control variables, respectively. In addition, *SP_i__t_* is social participation, *CV_i__t_* is the control variables and *ε_i__t_* is the error term.

Then this study used the decomposition method of the concentration index proposed by Wagstaff et al.^43^ to analyse the effect of social participation on income-related inequality in health outcome. The formula used for decomposition of the concentration index is as follows:

(4) \begin{equation*}C = \sum\nolimits_k {({\beta _k}{{\bar{x}}_k}} /\mu ){C_k} + G{C_\varepsilon }/\mu ,\end{equation*}

where *C* is the concentration index, *β_k_* is the marginal effect or coefficient of *x_k_, x_k_* is the mean of *x_k_, μ* is the mean of health outcome, *C_k_* is the concentration index for *x_k_* and *GC* is the generalized concentration index for *ε_i_*.

In this study, Stata 15.1 (StataCorp, College Station, TX, USA) was employed for descriptive statistics, the Hausman test, to calculate the concentration index, to construct the panel Logit regression model and panel linear regression model and to perform the decomposition of the concentration index. All tests were two-sided and p-values <0.05 were considered statistically significant.

## Results

### Descriptive statistics

Table 1 presents the descriptive statistics of all the variables used in this study from 2011 to 2014. In 2014, 58.33% of the respondents were IADL disabled. At the same time, the mean score of psychological health was 13.77, and >80% of respondents never participated in social activities. Furthermore, >42% of the respondents were ≥85 y of age. A roughly equal proportion of men and women were included in the sample, and approximately 47% of them were married. More than 98% of the respondents had <15 y of schooling. The mean household income increased from RMB 22 897.51 (US$3229.19) in 2011 to RMB 27 451.22 (US$3871.39) in 2014. In 2014, >89% of the respondents were covered by health insurance. In addition, >41% of the respondents lived in rural areas. Less than 38% of the respondents took part in physical exercise.

**Table 1.** Descriptive statistics

Variable	2011	2014
IADL status, n (%)		
IADL disabled	1160 (49.03)	1380 (58.33)
Have no IADL problem	1206 (50.97)	986 (41.67)
Psychological health		
Mean (SD)	14.00 (3.36)	13.77 (3.48)
Participation in social activities, n (%)		
No	1870 (79.04)	1899 (80.26)
Yes	496 (20.96)	467 (19.74)
Age (years), n (%)		
65–74	795 (33.60)	411 (17.37)
75–84	839 (35.46)	944 (39.90)
≥85	732 (30.94)	1011 (42.73)
Gender, n (%)		
Women	1189 (50.25)	1189 (50.25)
Men	1177 (49.75)	1177 (49.75)
Marital status, n (%)		
Single, divorced or widowed	1146 (48.44)	1254 (53.00)
Married	1220 (51.56)	1112 (47.00)
Years of schooling, n (%)		
0–14	2334 (98.65)	2334 (98.65)
≥15	32 (1.35)	32 (1.35)
Household income (RMB)		
Mean (SD)	22 897.51 (23 662.34)	27 451.22 (23 130.44)
Health insurance, n (%)		
No	302 (12.76)	258 (10.90)
Yes	2064 (87.24)	2108 (89.10)
Residency location, n (%)		
Rural area	1047 (44.25)	986 (41.67)
Urban area	1319 (55.75)	1380 (58.33)
Physical exercise, n (%)		
No	1271 (53.72)	1477 (62.43)
Yes	1095 (46.28)	889 (37.57)

### Income-related inequality in health outcome

**Figure 1.**
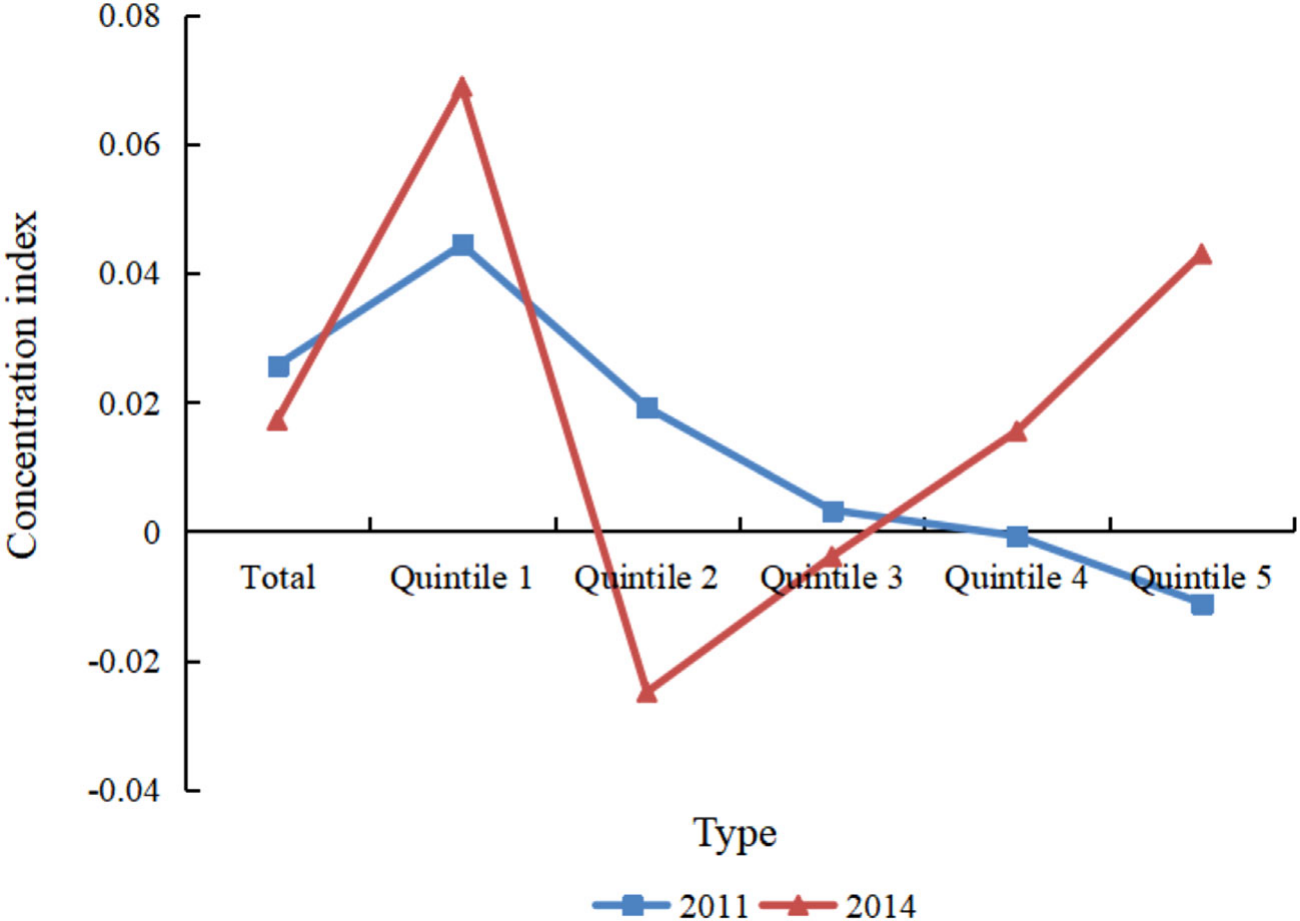
Concentration index of IADL status.

Figure 1 shows the concentration index of IADL status. The total concentration index values of IADL status from 2011 to 2014 were positive, indicating a pro-rich effect. In other words, the higher-income people were more likely to have no IADL problem than their lower-income counterparts. Furthermore, in 2014, the concentration index values of IADL status in quintiles 1, 4 and 5 were positive, which suggests that higher-income people tend to have no IADL problem, favouring the rich. At the same time, the concentration index values of IADL status in quintiles 2 and 3 were negative, suggesting that lower-income people tend to have no IADL problem, favouring the poor.

**Figure 2.**
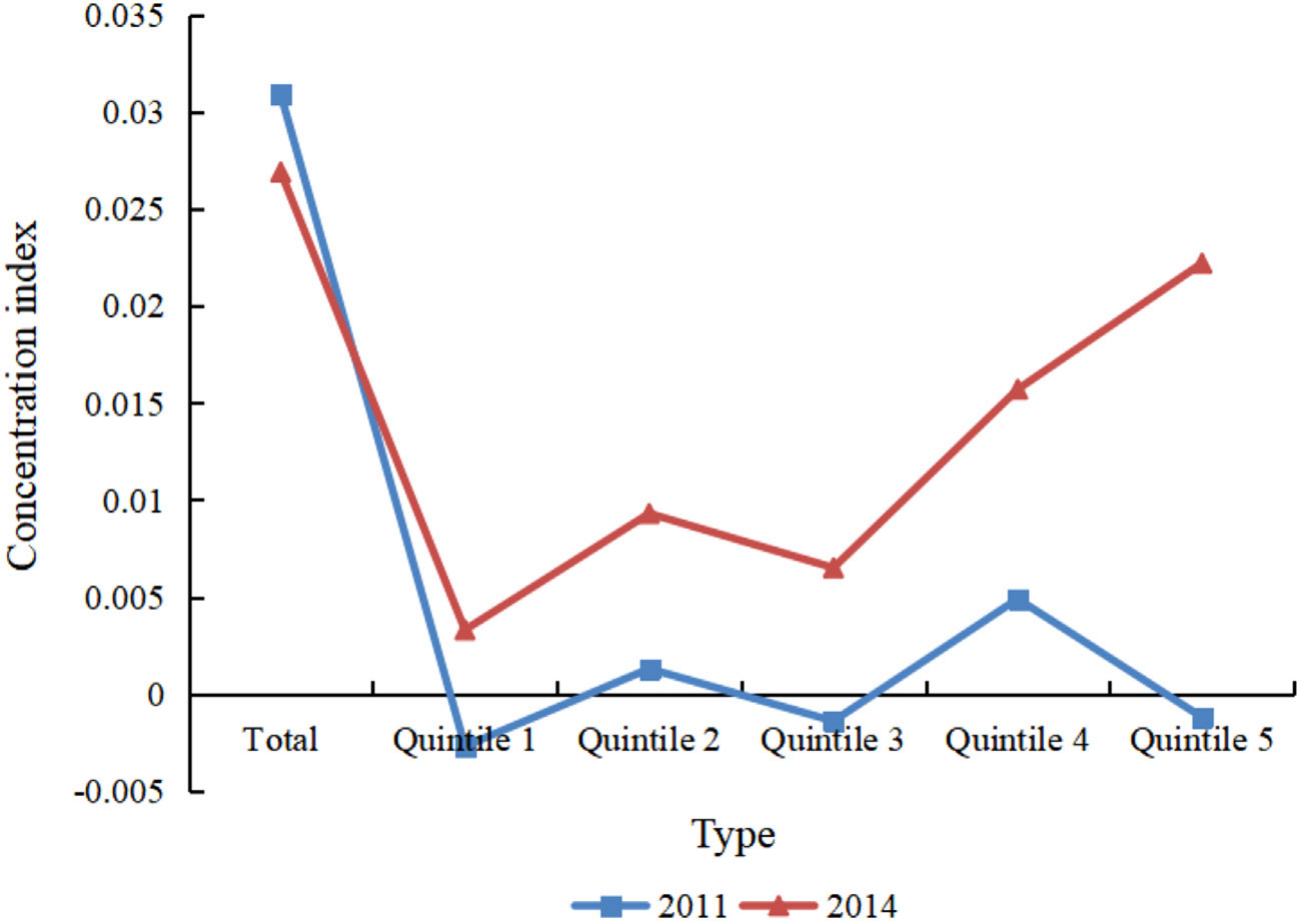
Concentration index of psychological health.

Figure 2 displays the concentration index of psychological health. The total concentration index values of psychological health from 2011 to 2014 were positive. In 2014, the concentration index values in quintiles 1–5 were positive, which reveals that higher-income people tend to have better psychological health, favouring the rich. The main reason may lie in the fact that higher-income people have more access to high-quality health services. Moreover, we also observed that the absolute values of the total concentration index for psychological health were larger than those for IADL status from 2011 to 2014, indicating a greater degree of income-related inequality in psychological health.

### The effect of social participation on health outcome

Table 2 shows the regression results of effects of social participation on IADL status and psychological health. In the first two columns we estimated the impact of social participation on IADL status. The Hausman test that compared coefficients of fixed effects and random effects results was statistically significant (p < 0.01), which indicates that the random effects results were biased and the fixed effects results were appropriate. The fixed effects results suggest that social participation significantly improved the probability of having no IADL problem by 42.04% among older adults (OR 1.4204, p < 0.05). This finding is consistent with the findings of Gao et al.^29^ and Hu et al.,^44^ who also found that social participation significantly reduced the risk of functional disability for Chinese older adults. Furthermore, taking the age group of 65–74 y as a reference, the probability of having no IADL problem was lower in the age group ≥85 y (OR 0.3440, p < 0.01). In addition, we also found that taking part in physical exercise (OR 1.8001, p < 0.01) could also significantly increase the probability of having no IADL problem.

**Table 2.** Regression results of effects of social participation on IADL status and psychological health

	IADL status		Psychological health	
Variable	Odds ratio	Standard error	Coefficient	Robust standard error
Participation in social activities				
No	Ref		Ref	
Yes	1.4204*	0.2250	0.4761**	0.1495
Age (years)				
65–74	Ref		Ref	
75–84	0.7226	0.1405	−0.3109	0.2030
≥85	0.3440**	0.1080	−0.7890*	0.3090
Gender				
Women	Ref		Ref	
Men	—	—	—	—
Marital status				
Single, divorced or widowed	Ref		Ref	
Married	1.4897	0.4307	0.3866	0.2847
Years of schooling	0.9732	0.0643	0.0757	0.0680
Household income	0.9080*	0.0432	0.0064	0.0489
Health insurance				
No	Ref		Ref	
Yes	0.8268	0.1592	0.1848	0.1895
Residency location				
Rural area	Ref		Ref	
Urban area	1.1072	0.1619	0.2235	0.1351
Physical exercise				
No	Ref		Ref	
Yes	1.8001**	0.2454	0.5823**	0.1456
Constant	—	—	13.1978**	0.5469
Model	Fixed effects		Fixed effects	
Hausman test	58.60**		64.34**	

Ref: reference group.

*p < 0.05, **p < 0.01.

Gender was omitted because of no within-group variance.

In the last two columns of Table 2 we estimated the effect of social participation on psychological health. The Hausman test was statistically significant (p < 0.01), which indicates that fixed effects results were appropriate. The fixed effects results indicate that social participation significantly improved the psychological health by 47.61% among older adults (coefficient = 0.4761, p < 0.01). This finding is consistent with the finding of Wen et al.,^45^ who discovered that participation in social activities significantly improved mental health. The reason is that participation in social activities can reduce loneliness and social isolation. Compared with the age group of 65–74 y, the age group ≥85 y had worse psychological health (coefficient = −0.7890, p < 0.05). Furthermore, it was also found that taking part in physical exercise (coefficient = 0.5823, p < 0.01) could significantly promote psychological health for the older adults.

### Effect of social participation on income-related inequality in IADL status

Table 3 provides the decomposition results of effect of social participation on income-related inequality in IADL status. The positive concentration index values of participation in social activities, residency location and physical exercise indicate that higher-income people are more likely to participate in social activities, live in an urban area and take part in physical exercise. In contrast, the concentration index value of having health insurance was negative, which reveals that health insurance is more concentrated among lower-income people.

**Table 3.** Decomposition results of effect of social participation on income-related inequality in IADL status

Variable	Elasticity	C_k_	Absolute contribution to C	Percentage contribution to C
Participation in social activities				
No	Ref			
Yes	0.2010	0.1276	0.0257	183.39
Age (years)				
65–74	Ref			
75–84	−0.5229	−0.0284	0.0149	106.25
≥85	−1.5960	0.0553	−0.0882	−630.91
Gender				
Women	Ref			
Men	0.8199	0.0110	0.0090	64.44
Marital status				
Single, divorced or widowed	Ref			
Married	0.2706	−0.0015	−0.0004	−2.89
Years of schooling	0.1738	0.1301	0.0226	161.61
Household income	0.1285	0.0821	0.0105	75.40
Health insurance				
No	Ref			
Yes	0.2979	−0.0054	−0.0016	−11.42
Residency location				
Rural area	Ref			
Urban area	−0.0269	0.0983	−0.0026	−18.90
Physical exercise				
No	Ref			
Yes	0.5618	0.1143	0.0642	459.10

C_k_: concentration index of the explanatory variable; C: concentration index of the independent variable; Ref: reference group.

The decomposition results indicate that social participation made a major contribution to the pro-rich inequality in IADL status, and the percentage contribution to the concentration index was 183.39%. The decomposition results also reveal that age ≥85 y (−630.91%) was identified as the biggest contributor to income-related inequality in IADL status. In addition, it was also found that physical exercise (459.10%) made a substantial contribution to the observed pro-rich inequality in IADL status. Except for the above, other factors such as years of schooling (161.61%), age 75–84 y (106.25%), household income (75.40%), gender (64.44%) and residency location (−18.90%) were also observed to make important contributions to the pro-rich inequality in IADL status.

### Effect of social participation on income-related inequality in psychological health

Table 4 shows the decomposition results of the effect of social participation on income-related inequality in psychological health. The decomposition results indicate that social participation made a minor contribution to the pro-rich inequality in psychological health, and the decomposed value was 3.41%. Xu et al.^26^ found that participation in social activities made a substantial contribution to the overall pro-rich inequality in depressive symptoms. Furthermore, the decomposition results also reveal that household income (49.02%), physical exercise (15.33%), years of schooling (5.44%) and residency location (4.27%) were identified as key factors to explain the pro-rich inequality in psychological health.

**Table 4.** Decomposition results of effect of social participation on income-related inequality in psychological health

Variable	Elasticity	C_k_	Absolute contribution to C	Percentage contribution to C
Participation in social activities				
No	Ref			
Yes	0.0075	0.1276	0.0010	3.41
Age (years)				
65–74	Ref			
75–84	0.0008	−0.0284	−2.25e-05	−0.08
≥85	0.0032	0.0553	0.0002	0.63
Gender				
Women	Ref			
Men	0.0116	0.0110	0.0001	0.46
Marital status				
Single, divorced or widowed	Ref			
Married	0.0174	−0.0015	−2.60e-05	−0.09
Years of schooling	0.0116	0.1301	0.0015	5.44
Household income	0.1662	0.0821	0.0136	49.02
Health insurance				
No	Ref			
Yes	0.0020	−0.0054	−1.08e-05	−0.04
Residency location				
Rural area	Ref			
Urban area	0.0121	0.0983	0.0012	4.27
Physical exercise				
No	Ref			
Yes	0.0373	0.1143	0.0043	15.33

C_k_: concentration index of the explanatory variable; C: concentration index of the independent variable; Ref: reference group.

## Discussion

This study examined the relationship between social participation and income-related inequality in health outcome among older adults using data from the 2011 and 2014 waves of the CLHLS. The results indicate that higher-income people tend to have no IADL problem. Furthermore, higher-income people are more likely to have better psychological health.

This study found that social participation significantly improved the IADL status among older adults. Furthermore, it was also found that social participation had a positive effect on psychological health. Moreover, we also obtained evidence indicating that social participation was observed to make a major contribution to the pro-rich inequality in IADL status. In addition, the decomposition analysis also revealed that social participation made a minor contribution to income-related inequality in psychological health.

These findings provide several important policy implications. First, it is necessary for the mass media to create a good environment for social participation of older adults and guide them to participate in social activities. Second, there is an urgent need for the government to strengthen the construction of communities and provide more funding to organize a variety of social activities. Third, the government needs to develop associations of older adults to promote and maintain social participation. Finally, older adults should actively take part in social activities in order to improve health outcomes.

This study has several strengths. To the best of our knowledge, this is the first study to use a national representative sample to investigate the effect of social participation on income-related inequality in health outcomes among older adults in the context of China. Furthermore, this study employed IADL status and psychological health to measure health outcome, which helps comprehensively assess the health effect of social participation among Chinese older adults. Moreover, our study provides empirical support for the notion that interventions to promote social participation can lead to the improvement in health outcomes among older adults.

However, it must be acknowledged that this study has two limitations. Given that the data used in this study are self-reported, recall bias may exist. Also, air pollution has been discovered to produce a significant effect on health outcome,^46^ but we cannot control for it with these data. Therefore future studies should control for this factor.

## Conclusions

In conclusion, this study demonstrated that there were overall pro-rich inequalities in IADL status and psychological health among older adults in China. Furthermore, social participation had beneficial effects on IADL status and psychological health. Moreover, social participation made a major contribution to the pro-rich inequality in IADL status, while it made a minor contribution to the pro-rich inequality in psychological health. This study highlights the importance of focusing on promoting social participation when formulating health policy aimed at improving healthy ageing.
